# Renal Function as a Predictor of Mortality and Functional Outcomes in Acute Stroke: A Prospective Study

**DOI:** 10.7759/cureus.73176

**Published:** 2024-11-06

**Authors:** Deekshitha Reddy Y, Vijayashree Thyagaraj, Viraj Shetty, Rizwanullah, Albine Djeagou, Sophia Tahir, Satkarjeet Kaur Gill, Muhammad Usman Khan, Aftab Ahmad, Henna Patel

**Affiliations:** 1 General Medicine, MS Ramaiah Medical College, Bengaluru, IND; 2 Internal Medicine, MS Ramaiah Medical College, Bengaluru, IND; 3 Medicine, Victoria Hospital, Bengaluru, IND; 4 Internal Medicine, Hayatabad Medical Complex Peshawar, Peshawar, PAK; 5 Faculty of Health Sciences, University of Buea, Buea, CMR; 6 Internal Medicine, Windsor University School of Medicine, Cayon, KNA; 7 Internal Medicine, Jagare Ridge Medical Clinic, Edmonton, CAN; 8 Internal Medicine, Cumberland Infirmary, Carlisle, GBR; 9 General Medicine, Cork University Hospital, Cork, IRL; 10 Medicine, GMERS (Gujarat Medical Education and Research Society) Medical College, Vadodara, IND

**Keywords:** egfr, functional outcomes, hemorrhagic stroke, ischemic stroke, modified rankin scale, mortality predictors, mrs, renal function, stroke

## Abstract

Background: Stroke is a leading cause of global morbidity and mortality, and understanding modifiable predictors of stroke outcomes is crucial. This study explores the relationship between renal function, assessed via estimated glomerular filtration rate (eGFR) and serum creatinine, and stroke mortality and functional outcomes in a cohort of acute stroke patients in India.

Methods: A prospective observational study was conducted from February 2021 to October 2022, including 104 acute stroke patients from MS Ramaiah Medical College and Hospital, Bengaluru, India. Patients were categorized into ischemic and hemorrhagic stroke groups. Data collected included demographic information, clinical history, and laboratory results. Functional outcomes were measured using the Modified Rankin Scale (mRS) at admission, day 7, and discharge. Renal function was evaluated using serum creatinine and eGFR. Statistical analyses, including multiple logistic regression, were performed to identify predictors of mortality.

Results: The mean age of participants was 62.2 years, with a male predominance of 75 (72.1%). Hemorrhagic stroke patients had higher mortality rates compared to ischemic stroke patients (p = 0.006). Elevated serum creatinine and decreased eGFR were significantly associated with poorer outcomes. The eGFR at admission was lower in hemorrhagic stroke patients (55.6 ± 34.2 mL/min) compared to ischemic stroke patients (67.3 ± 29.6 mL/min; p = 0.048). Multiple logistic regression revealed that hemorrhagic stroke (OR = 0.17; 95% CI: 0.06 - 0.50) and age (OR = 1.06; 95% CI) were independent predictors of mortality. The threshold values for predicting mortality risk were 1.495 mg/dL for serum creatinine (sensitivity: 69.6%, specificity: 81.8%) and 48.5 mL/min for eGFR (sensitivity: 86.4%, specificity: 69.5%). These thresholds exhibited strong performance in assessing mortality risk, with AUCs (Area Under the Curve) of 0.765 for serum creatinine and 0.786 for eGFR.

Conclusion: Renal function indicators, specifically serum creatinine, and eGFR, are significant predictors of mortality and functional outcomes in acute stroke patients. Hemorrhagic stroke is associated with higher mortality and poorer renal function compared to ischemic stroke. These findings highlight the importance of assessing renal function in stroke management and suggest further research with larger cohorts to refine predictive models for stroke outcomes.

## Introduction

According to the World Health Organization (WHO), stroke is defined as a sudden onset of clinical signs of focal (or global) disruption of cerebral function, lasting more than 24 hours or resulting in death, with no apparent cause other than a vascular origin [1]. Stroke is a major global health burden, ranking as the second-leading cause of mortality and the third-leading cause of combined mortality and disability, as measured by disability-adjusted life-years (DALYs) lost [2]. In India, where both communicable and non-communicable diseases are prevalent, the estimated adjusted stroke prevalence ranges from 84 to 262 per 100,000 in rural areas and from 334 to 424 per 100,000 in urban areas, with an incidence rate of 119 to 145 per 100,000 based on recent studies [3].

Identifying modifiable predictors of stroke mortality is crucial to improving patient outcomes through early intervention. While risk factors like stroke severity, age, sex, type of stroke, and consciousness level are significant, they are mostly non-modifiable, limiting their utility in clinical practice [4].

Acute kidney injury (AKI) is defined by an increase in serum creatinine by 0.3 mg/dl within 48 hours, a 1.5-fold rise in serum creatinine from baseline within seven days, or urine output below 0.5 ml/kg/h for six hours [5]. Renal dysfunction, marked by an estimated glomerular filtration rate (eGFR) of less than 60 mL/min/1.73 m², presents a significant risk of cardiovascular morbidity and mortality [5]. Studies have shown a graded relationship between declining eGFR and higher risks of death, cardiovascular events, and hospitalizations [6]. In a cohort study of 2,042 stroke patients, renal markers such as serum creatinine and urea at admission were strong predictors of mortality, even after adjusting for traditional risk factors such as age, neurological status, and comorbidities (e.g., congestive heart failure, ischemic heart disease, hypertension, and smoking) [7]. Additionally, a meta-analysis involving 284,672 patients highlighted that renal dysfunction (eGFR <60 mL/min/1.73 m²) independently increases the risk of stroke [8].

The modified Rankin Scale (mRS) is a seven-level tool that offers several advantages. It comprehensively captures the range of functional outcomes, from no symptoms to death. The categories are straightforward, making it easy for both patients and healthcare providers to comprehend. Its validity is supported by strong correlations with stroke pathology indicators, like infarct size, and its alignment with other stroke assessment scales. Moreover, the mRS has been instrumental in differentiating effective from ineffective acute stroke treatments. Although it has fewer levels than some other stroke assessments, even a one-point change in the mRS holds clinical importance [9,10].

Despite these findings, there remains a lack of comprehensive analysis on how renal impairment influences stroke incidence and prognosis. Given stroke's vascular nature, understanding the role of renal function in overall cardiovascular risk post-stroke is vital for developing preventive and therapeutic strategies.

## Materials and methods

A prospective observational study was conducted at the Departments of Medicine and Neurosciences at MS Ramaiah Medical College and Hospital, Bengaluru, India, from February 2021 to October 2022, involving 104 acute stroke patients. The Ethical Committee, MS Ramaiah Medical College and Hospital, Bengaluru issued approval MSRMC/EC/PG-28/01-2021. The study included adults above 18 years with stroke confirmed by CT/MRI, excluding those with transient ischemic attacks, late presentations (>72 hours), previous stroke history, or strokes caused by tuberculoma, tumors, trauma, or known renal/hepatic disease. Data collected included demographic information, medical history, physical examination, blood pressure measurements, and various laboratory investigations such as serum creatinine, lipid profile, and blood sugar. Serum creatinine was measured using the Jaffe method, and outcomes were assessed using the mRS on day 0, day 7, and at discharge. The eGFR was calculated using the CKD-EPI equation.

The primary objectives were to assess the association between eGFR at admission and the functional outcomes of ischemic and hemorrhagic stroke, as well as to evaluate the role of renal function in hospitalized stroke patients. Based on previous research linking eGFR with mortality and functional outcomes in stroke patients [11], a sample size of 104 was determined to ensure adequate statistical power.

Statistical analysis was performed using R software (version 4.4.2, R Foundation for Statistical Computing, Vienna, Austria). Continuous variables were described using mean, standard deviation, median, and interquartile range, while categorical variables were described using frequencies and percentages. The Shapiro-Wilk test was used to test the normality of continuous variables, and these were compared using either the t-test or the non-parametric Kruskall-Wallis test for non-normally distributed variables. The Fisher-Exact test was used to compare categorical variables. Factors associated with mortality in stroke patients were assessed by first performing bivariate analyses, and then identifying variables with p-values <0.2. Multiple logistic regression was performed to identify those factors that were independently associated with the risk of death. Variables selection was done in a backward stepwise mode, and then gradually deleted variables with the highest p-values. Model performance was evaluated using the “R-squared; R2”. Nested models were tested by using the likelihood ratio test. The level of statistical significance was set at 5%.

## Results

Characteristics on admission

Baseline Characteristics and Other Co-morbidities

The mean age of the study population was 62.2 years, with a predominance of males 75 (72.1%), and no statistically significant difference in age or gender distribution between the types of stroke. Patients with hemorrhagic stroke were more likely to have a history of hypertension compared to those with ischemic stroke (p = 0.026). Other baseline characteristics were not significantly different between the two stroke types (Table 1).

**Table 1:** Baseline characteristics and co-morbidities. SD; Standard deviation, Min; Minimum, Max; Maximum.

Variables	Statistics	Total	Hemorrhagic	Ischemic	p-value
Age (years)	N	104	30	74	0.193
	Mean(SD)	62.2(13.6)	59.9(10.7)	63.1(14.5)	
	Min-max	26.0-89.0	39.0-82.0	26.0-89.0	
Sex	N	104	30	74	0.676
	Male (%)	75 (72.1)	23 (76.7)	52 (70.3)	
	Female (%)	29 (27.9)	7 (23.3)	22 (29.7)	
Heart failure	N	104	30	74	0.322
	No (%)	100 (96.2)	30 (100.0)	70 ( 94.6)	
	Yes (%)	4 (3.8)	0 (0.0)	4 (5.4)	
Hypertension	N	104	30	74	0.026
	No (%)	28 (26.9)	3 (10.0)	25 (33.8)	
	Yes (%)	76 (73.1)	27 (90.0)	49 (66.2)	
Diabetes	N	104	30	74	1
	No (%)	54 (51.9)	16 (53.3)	38 (51.4)	
	Yes (%)	50 (48.1)	14 (46.7)	36 (48.6)	
Alcohol	N	104	30	74	0.401
	No (%)	83 (79.8)	26 (86.7)	57 (77.0)	
	Yes (%)	21 (20.2)	4 (13.3)	17 (23.0)	
Smoking	N	104	30	74	0.317
	No (%)	78 (75.0)	25 (83.3)	53 (71.6)	
	Yes (%)	26 (25.0)	5 (16.7)	21 (28.4)	
Thyroid disease	N	104	30	74	1
	No (%)	99 (95.2)	29 (96.7)	70 (94.6)	
	Yes (%)	5 (4.8)	1 (3.3)	4 (5.4)	
Atrial fibrillation	N	104	30	74	1
	No (%)	97 (93.3)	28 (93.3)	69 (93.2)	
	Yes (%)	7 (6.7)	2 (6.7)	5 (6.8)	
Ischemic heart disease	N	104	30	74	0.752
	No (%)	90 (86.5)	27 (90.0)	63 (85.1)	
	Yes (%)	14 (13.5)	3 (10.0)	11 (14.9)	
Other medical conditions	N	104	30	74	0.692
	No (%)	86 (82.7)	26 (86.7)	60 (81.1)	

Laboratory Findings 

Patients with ischemic stroke had significantly higher LDL levels compared to those with hemorrhagic stroke (p = 0.04). In contrast, total leukocyte count (TLC) and creatinine levels were notably elevated in hemorrhagic stroke cases (p = 0.038 and p = 0.026, respectively). Additionally, eGFR values were lower in patients with hemorrhagic stroke (p = 0.048) (Table 2).

**Table 2:** Comparison of laboratory findings in ischemic versus hemorrhagic stroke patients. SD; Standard deviation, Min; Minimum, Max; Maximum, TLC; Total leukocyte count, eGFR; Estimated glomerular filtration rate, VLDL; Very low-density lipoprotein, LDL; Low-density lipoprotein, HDL; High-density lipoprotein, RBS; Random blood sugar, gm/dL; gram per deciliter, 10^3 /µl; cubic per microliter, mg/dL; milligram per deciliter, mEq/L; milliequivalents per liter.

Variables	Statistics	Total	Hemorrhagic	Ischemic	p-value
Hemoglobin (g/dl)	N	104	30	74	0.98
	Mean(SD)	13.2(2.4)	13.1(2)	13.2(2.6)	
	Min-Max	5.2-19.7	8.2-16.2	5.2-19.7	
Platelets	N	104	30	74	0.3
	Mean(SD)	256474(94702.2)	243866.7(109540)	261585.1(88293.1)	
	Min-Max	31300.0-601000.0	54000.0-601000.0	31300.0-584000.0	
TLC	N	104	30	74	0.038
	Mean(SD)	11976.9(4334.5)	13125.7(4421.3)	11511.2(4240.4)	
	Min-Max	4000.0-32100.0	4000.0-24900.0	6000.0-32100.0	
Creatinine (mg/dl)	N	104	30	74	0.026
	Mean(SD)	1.5(0.9)	1.9(1.4)	1.3(0.6)	
	Min-Max	0.5-6.9	0.5-6.9	0.5-3.2	
eGFR	N	104	30	74	0.048
	Mean(SD)	63.9(31.3)	55.6(34.2)	67.3(29.6)	
	Min-Max	9.0-129.0	9.0-110.0	14.0-129.0	
Total cholesterol	N	101	27	74	0.207
	Mean(SD)	164.8(41)	154.9(38.6)	168.4(41.5)	
	Min-Max	65.0-260.0	81.0-218.0	65.0-260.0	
	Missing	3	3	0	
Triglycerides	N	101	27	74	0.256
	Mean(SD)	135.9(59.6)	121.1(36.8)	141.3(65.3)	
	Min-Max	54.0-469.0	54.0-206.0	56.0-469.0	
	Missing	3	3	0	
VLDL	N	100	27	73	0.509
	Mean(SD)	25.7(9.6)	23.8(7.4)	26.4(10.2)	
	Min-Max	11.0-55.0	11.0-43.0	11.0-55.0	
	Missing	4	3	1	
LDL	N	101	27	74	0.04
	Mean(SD)	92.6(34.7)	79.4(27.8)	97.4(35.8)	
	Min-Max	22.0-187.0	22.0-131.0	26.0-187.0	
	Missing	3	3	0	
HDL	N	101	27	74	0.456
	Mean(SD)	40.8(9.6)	41.4(6.3)	40.6(10.6)	
	Min-Max	16.0-75.0	30.0-58.0	16.0-75.0	
	Missing	3	3	0	
RBS	N	104	30	74	0.514
	Mean(SD)	190.2(77.2)	199.8(87.7)	186.3(72.8)	
	Min-Max	88.0-412.0	88.0-412.0	95.0-385.0	
Sodium	N	104	30	74	0.485
	Mean(SD)	136.1(5.4)	136.5(4.6)	135.9(5.6)	
	Min-Max	113.0-152.0	127.0-148.0	113.0-152.0	
Potassium	N	104	30	74	0.466
	Mean(SD)	4.3(0.6)	4.4(0.6)	4.3(0.5)	
	Min-Max	3.3-5.7	3.3-5.5	3.5-5.7	

Stroke outcome

Functional Stroke Outcome

**Figure 1:**
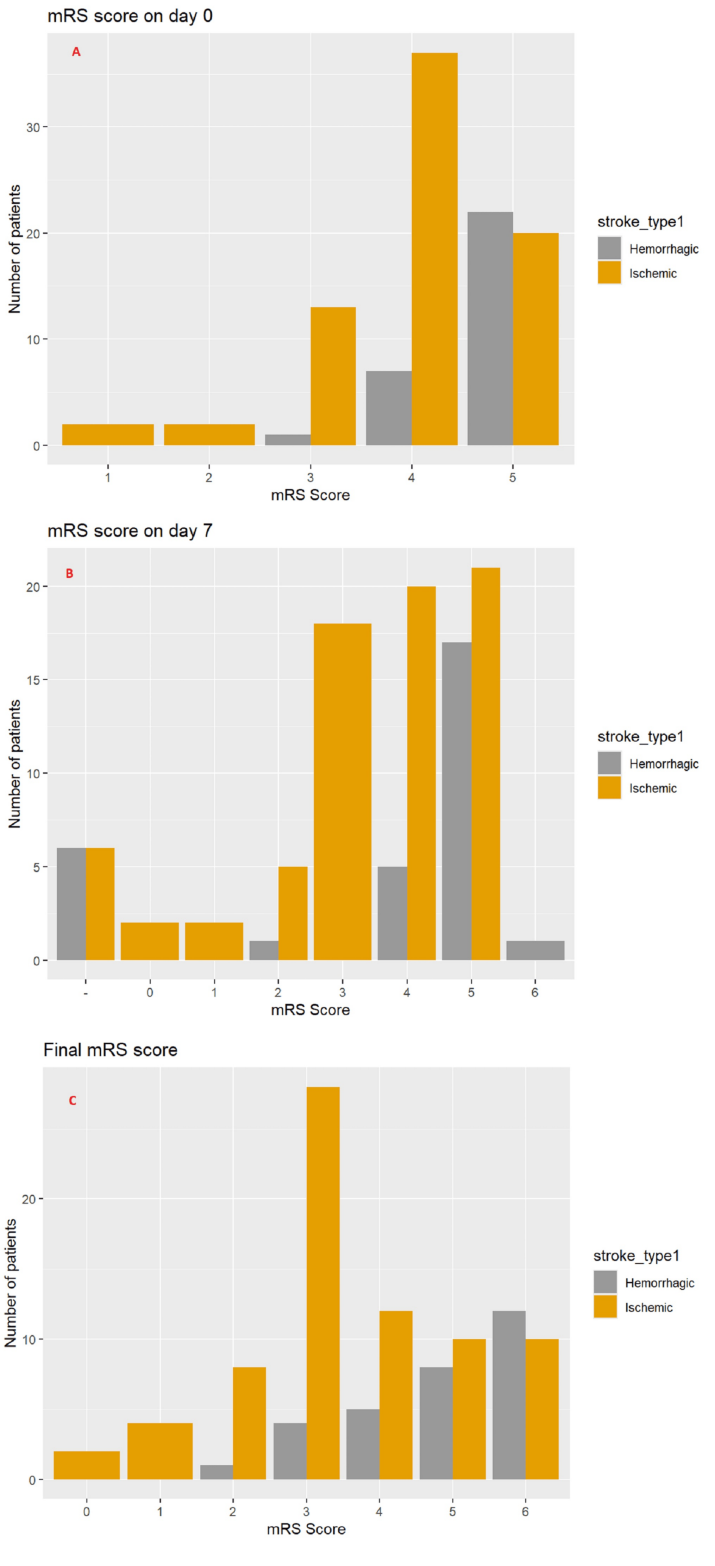
Measurement of modified Rankin Scale (mRS) on day 0, 7 and on discharge (A, B and C respectively) to measure functional outcome of stroke.

The modified Rankin Scale (mRS) was assessed at admission, on the 7th day, and at discharge to evaluate the functional outcome of stroke patients. Despite the limited duration of follow-up, the results indicated an overall improvement in functional status from admission to discharge. Hemorrhagic stroke patients exhibited higher mortality rates, while those with ischemic stroke presented with more severe initial symptoms (higher mRS scores). However, ischemic stroke patients demonstrated a faster recovery compared to hemorrhagic stroke patients, reflecting a better functional outcome by discharge (Figure 1).

Stroke Mortality

The duration of hospital stay was longer for hemorrhagic stroke patients, but this difference was not statistically significant (p = 0.148). However, mortality rates were significantly higher in hemorrhagic stroke patients compared to ischemic stroke patients (p = 0.006) (Table 3).

**Table 3:** Duration of hospital stay and outcome of stroke. STD; Standard deviation, Min; Minimum, Max; Maximum.

Variables	Statistics	Total	Hemorrhagic	Ischemic	p-value
Duration of hospital stay (days)	N	104	30	74	0.148
	Mean(SD)	15.6(10.8)	19.2(15.7)	14.1(7.7)	
	Min-Max	2.0-76.0	2.0-76.0	3.0-41.0	
Outcome	N	104	30	74	0.006
	Deceased (%)	22 (21.2)	12 (40.0)	10 (13.5)	
	Discharged (%)	82 (78.8)	18 (60.0)	64 (86.5)	

Factors Associated With Mortality

Multiple logistic regression analysis was employed to identify factors independently associated with mortality in patients admitted with stroke. Variables with a p-value ≤ 0.2 in the bivariate analysis were initially included in the model, followed by a backward stepwise selection process. The final model identified the type of stroke (ischemic versus hemorrhagic) and age as independent predictors of mortality.

Patients with ischemic stroke had a 17% lower likelihood of death compared to those with hemorrhagic stroke (OR = 0.17; 95% CI: 0.06 - 0.50). Additionally, each year increase in age was associated with a 6% increase in the odds of mortality from stroke (OR = 1.06; 95% CI) (Table 4).

**Table 4:** Multiple logistic regression analysis of factors associated with mortality in stroke patients. SD; Standard deviation, Min; Minimum, Max; Maximum, BP; blood pressure, TLC; Total leukocyte count, VLDL; Very low-density lipoprotein, LDL; Low-density lipoprotein, HDL; High-density lipoprotein, RBS; Random blood sugar, gm/dL; gram per deciliter, mg; milligram per deciliter.

Variables	Statistics	Total	Deceased	Discharged	p-value
Type of stroke	N	104	22	82	0.006
	Hemorrhagic (%)	30 (28.8)	12 (54.5)	18 (22.0)	
	Ischemic (%)	74 (71.2)	10 (45.5)	64 (78.0)	
Age (years)	N	104	22	82	0.073
	Mean(SD)	62.2(13.6)	67(11.1)	61(13.9)	
	Min-Max	26.0-89.0	47.0-89.0	26.0-89.0	
Sex	N	104	22	82	0.845
	Male (%)	75 (72.1)	15 (68.2)	60 (73.2)	
	Female (%)	29 (27.9)	7 (31.8)	22 (26.8)	
Heart failure	N	104	22	82	1
	No (%)	100 (96.2)	21 (95.5)	79 (96.3)	
	Yes (%)	4 (3.8)	1 (4.5)	3 (3.7)	
Hypertension	N	104	22	82	0.19
	No (%)	28 (26.9)	3 (13.6)	25 (30.5)	
	Yes (%)	76 (73.1)	19 (86.4)	57 (69.5)	
Diabetes	N	104	22	82	0.318
	No (%)	54 (51.9)	14 (63.6)	40 (48.8)	
	Yes (%)	50 (48.1)	8 (36.4)	42 (51.2)	
Alcohol	N	104	22	82	0.553
	No (%)	83 (79.8)	19 (86.4)	64 (78.0)	
	Yes (%)	21 (20.2)	3 (13.6)	18 (22.0)	
Smoking	N	104	22	82	1
	No (%)	78 (75.0)	16 (72.7)	62 (75.6)	
	Yes (%)	26 (25.0)	6 (27.3)	20 (24.4)	
Thyroid disease	N	104	22	82	0.285
	No (%)	99 (95.2)	20 (90.9)	79 (96.3)	
	Yes (%)	5 (4.8)	2 (9.1)	3 (3.7)	
Atrial fibrillation	N	104	22	82	0.162
	No (%)	97 (93.3)	19 (86.4)	78 (95.1)	
	Yes (%)	7 (6.7)	3 (13.6)	4 ( 4.9)	
Ischemic heart disease	N	104	22	82	0.488
	No (%)	90 (86.5)	18 (81.8)	72 (87.8)	
	Yes (%)	14 (13.5)	4 (18.2)	10 (12.2)	
Other medical conditions	N	104	22	82	0.058
	No (%)	86 (82.7)	15 (68.2)	71 (86.6)	
	Yes (%)	18 (17.3)	7 (31.8)	11 (13.4)	
Pulse rate	N	104	22	82	0.261
	Mean(SD)	86.6(16.2)	91(20.5)	85.5(14.7)	
	Min-Max	60.0-170.0	66.0-164.0	60.0-170.0	
Diastolic BP (mmHg)	N	104	22	82	0.797
	Mean(SD)	92(16)	90.9(21.4)	92.3(14.4)	
	Min-Max	50.0-150.0	50.0-150.0	70.0-140.0	
Systolic BP (mmHg)	N	104	22	82	0.835
	Mean(SD)	159.2(34)	155.9(41.4)	160.1(32)	
	Min-Max	80.0-240.0	80.0-230.0	110.0-240.0	
Duration of hospital stay (days)	N	104	22	82	0.02
	Mean(SD)	15.6(10.8)	11.8(9.3)	16.6(11)	
	Min-Max	2.0-76.0	2.0-40.0	5.0-76.0	
Hemoglobin(g/dl)	N	104	22	82	0.35
	Mean(SD)	13.2(2.4)	12.8(2.4)	13.3(2.4)	
	Min-Max	5.2-19.7	8.2-16.9	5.2-19.7	
Platelets	N	104	22	82	0.984
	Mean(SD)	256474(94702.2)	264545.5(126305.6)	254308.5(85123.3)	
	Min-Max	31300.0-601000.0	54000.0-601000.0	31300.0-584000.0	
TLC	N	104	22	82	0.225
	Mean(SD)	11976.9(4334.5)	12987.7(4662.6)	11705.7(4231.1)	
	Min-Max	4000.0-32100.0	6000.0-24900.0	4000.0-32100.0	
Creatinine (mg/dl)	N	104	22	82	<0.0001
	Mean(SD)	1.5(0.9)	2.1(1.3)	1.3(0.7)	
	Min-Max	0.5-6.9	0.6-6.9	0.5-4.0	
Estimated glomerular filtration rate	N	104	22	82	<0.0001
	Mean(SD)	63.9(31.3)	39.5(22.3)	70.5(30.2)	
	Min-Max	9.0-129.0	9.0- 99.0	14.0-129.0	
Total cholesterol	N	101	22	79	0.104
	Mean(SD)	164.8(41)	149(40.3)	169.2(40.3)	
	Min-Max	65.0-260.0	65.0-224.0	93.0-260.0	
	Missing	3	0	3	
Triglycerides	N	101	22	79	0.086
	Mean(SD)	135.9(59.6)	116.6(36)	141.2(63.8)	
	Min-Max	54.0-469.0	54.0-199.0	56.0-469.0	
	Missing	3	0	3	
VLDL	N	100	22	78	0.029
	Mean(SD)	25.7(9.6)	21.6(6.5)	26.9(10)	
	Min-Max	11.0-55.0	11.0-43.0	11.0-55.0	
	Missing	4	0	4	
LDL	N	101	22	79	0.026
	Mean(SD)	92.6(34.7)	76.5(28.1)	97.1(35.1)	
	Min-Max	22.0-187.0	22.0-122.0	37.0-187.0	
	Missing	3	0	3	
HDL	N	101	22	79	0.821
	Mean(SD)	40.8(9.6)	41(10.3)	40.8(9.4)	
	Min-Max	16.0-75.0	18.0-74.0	16.0-75.0	
RBS	N	104	22	82	0.368
	Mean(SD)	190.2(77.2)	185.4(92.8)	191.5(73.1)	
	Min-Max	88.0-412.0	88.0-379.0	95.0-412.0	
Sodium	N	104	22	82	0.22
	Mean(SD)	136.1(5.4)	136.8(8)	135.9(4.5)	
	Min-Max	113.0-152.0	113.0-149.0	126.0-152.0	
Potassium	N	104	22	82	0.003
	Mean(SD)	4.3(0.6)	4.6(0.5)	4.2(0.6)	
	Min-Max	3.3-5.7	3.7-5.5	3.3-5.7	

Diagnostic potential of renal function in predicting the risk of death among stroke patients

Creatinine

**Figure 2:**
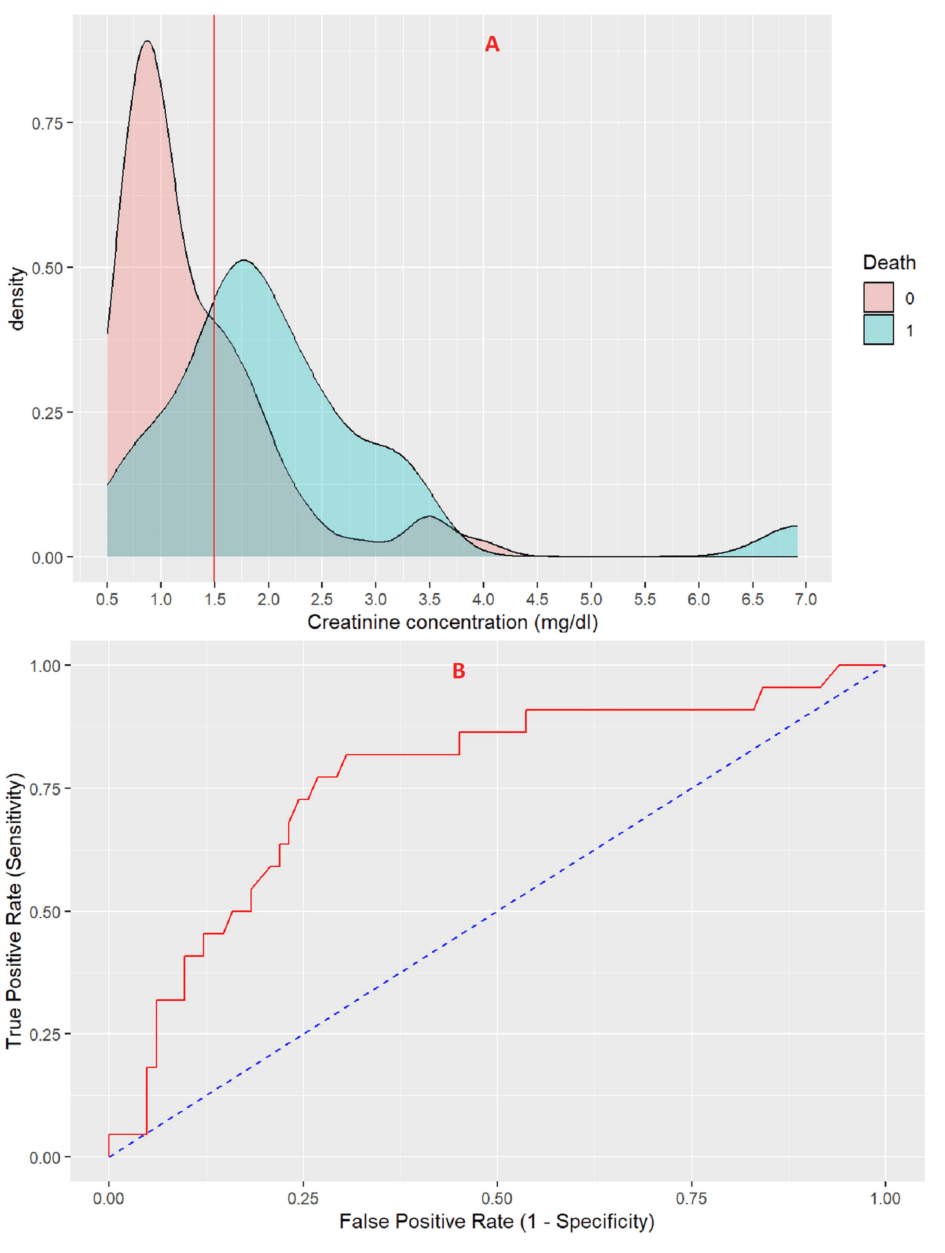
(A) Density plot of creatinine concentration, (B) ROC curve for risk of death in stroke patient determine by creatinine ROC; Receiver operating characteristic

The threshold value of creatinine concentration for predicting mortality risk in stroke patients is 1.495 mg/dl (Figure 2A). At this threshold, the creatinine level demonstrates a sensitivity of 69.6% and a specificity of 81.8%. The positive predictive value is 93.4%, while the negative predictive value is 41.9%. The creatinine level shows a strong performance in identifying death risk among stroke patients, with an area under the curve (AUC) of 0.765 (95% CI: 0.648 - 0.882) (Figure 2B).

Estimated Glomerular Filtration Rate (eGFR)

**Figure 3:**
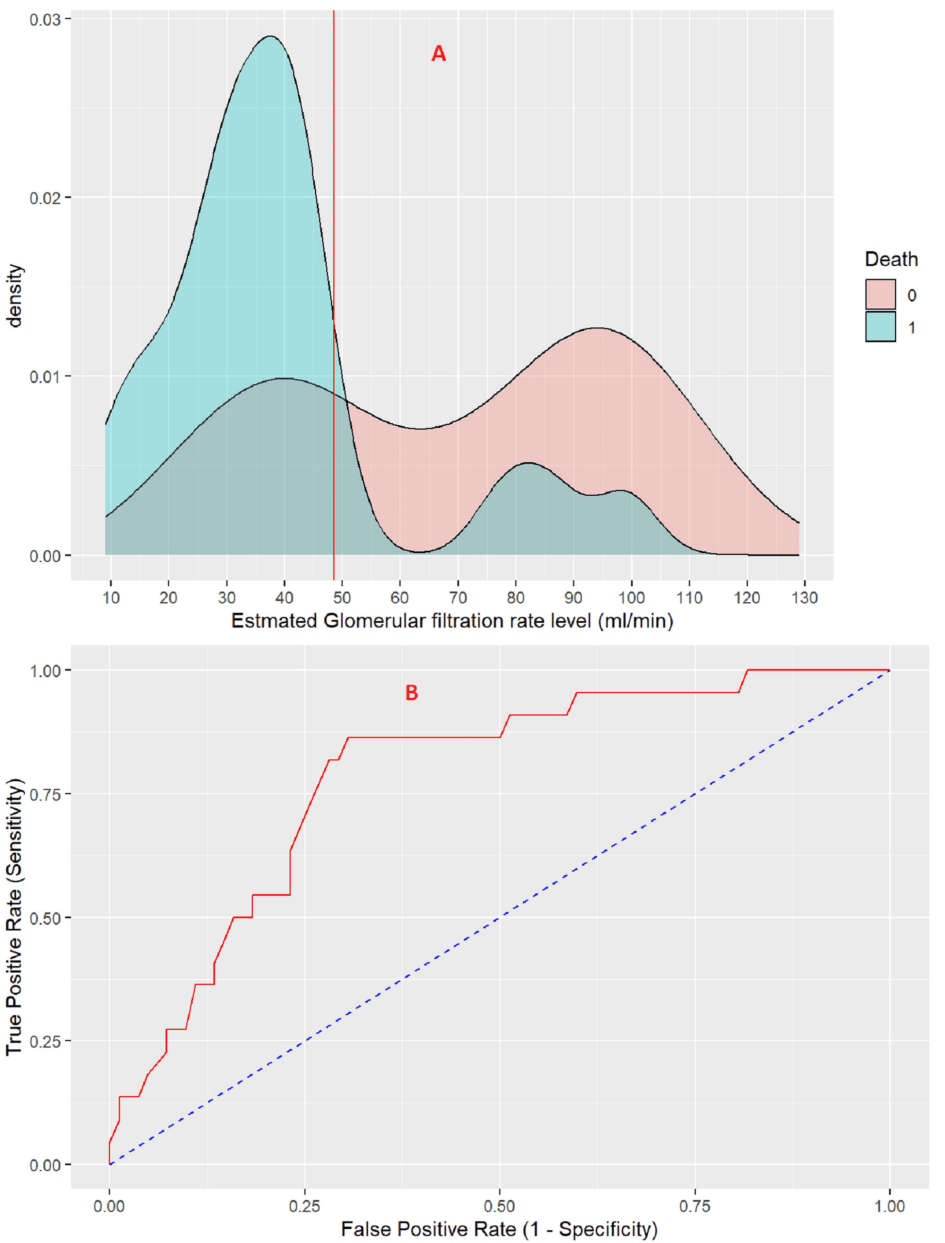
(A) Density plot of eGFR, (B) ROC curve for risk of death in stroke patient determine by eGFR. eGFR; Estimated glomerular filtration rate, ROC; Receiver operating characteristic.

The threshold value of the eGFR for predicting mortality risk in stroke patients is 48.5 ml/min (Figure 3A). At this threshold, the eGFR exhibits a sensitivity of 86.4% and a specificity of 69.5%. The positive predictive value is 43.2%, while the negative predictive value is 95.0%. The eGFR demonstrates strong performance in assessing the risk of death in stroke patients, with an area under the curve (AUC) of 0.786 (95% CI: 0.6848 - 0.8873) (Figure 3B).

## Discussion

In our study, the average age of participants was 62.2 years, with 75 (72.1%) being male. Age and gender distribution between stroke types showed no significant difference (P = 0.193 and P = 0.676, respectively). Wyller TB reported higher stroke incidence in men (372 vs. 340 per 100,000), with women experiencing their first stroke at a later age than men (median age 72.0 vs. 66.5 years, P = 0.013) [12]. Similarly, Nagaraja D et al. found that men made up (786) 67% of stroke cases in a cohort of 1,174 patients in Bangalore [13]. In a study, it has been noted that the average age of stroke onset in India is 63 years, younger than in Western countries, where the onset is 68 years in the USA and 71 years in Italy [11]. A meta-analysis by Rahbar MH et al., which included 67 studies, determined the mean age of stroke to be 64.4 years (95% CI: 62.9, 65.8) [14].

A study from North India identified key stroke risk factors such as hypertension (diastolic blood pressure >95 mmHg), hyperglycemia, tobacco use, and low hemoglobin (<10 g%) [15]. Another community-based study highlighted the association of elevated fasting blood glucose, cholesterol, triglycerides, and low HDL levels with increasing urbanization [16]. India faces a dual burden of tobacco exposure, with 15-20% of the population smoking and up to 40% using smokeless tobacco [17].

Our study revealed significantly lower eGFR in hemorrhagic stroke patients compared to ischemic stroke patients (Mean (STD) = 55.6 (34.2) vs. 67.3 (29.6), P = 0.048). This finding aligns with another study that reported lower eGFR in hemorrhagic stroke patients than in ischemic stroke patients (64.79 ± 25.85 vs. 86.04 ± 26.09, P = 0.005) [18].

In our study, 30 (29%) experienced hemorrhagic strokes, while 74 (71%) suffered ischemic strokes. Similarly, the Indian Collaborative Acute Stroke Study (ICASS) found that 1665 (77%) of stroke cases were ischemic, 476 (22%) hemorrhagic, and 22 (1%) unspecified based on cerebral CT scans [19]. Globally, Feigin VL et al. reported that ischemic strokes comprised 62.4% of all stroke cases, while intracerebral hemorrhages accounted for 27.9% and subarachnoid hemorrhages 9.7% [20].

Hypertension was the only comorbidity significantly associated with hemorrhagic stroke in comparison to ischemic stroke (P = 0.026). No significant differences were found for other baseline characteristics such as atrial fibrillation, alcohol use, smoking history, diabetes, ischemic heart disease (IHD), heart failure, or dyslipidemia. Another study found that 28.8% of hemorrhagic stroke patients had stage 2 hypertension, and 15.9% had stage 3 hypertension [21].

Patients with ischemic stroke in our study exhibited significantly higher LDL levels than those with hemorrhagic stroke (P = 0.04). This finding mirrors other studies, which reported elevated serum low-density lipoprotein (LDL) in ischemic stroke patients [22]. On the other hand, total leukocyte count (TLC) and creatinine levels were significantly elevated in hemorrhagic stroke patients (P = 0.038 and P = 0.026, respectively). For ischemic stroke patients, impaired consciousness, elevated TLC, increased ESR, and raised creatinine and SGPT (serum glutamic-pyruvic transaminase) levels within 24 hours of hospitalization were key indicators of 30-day mortality [23].

Lower eGFR was more prevalent in hemorrhagic stroke patients (P = 0.048), who also experienced a higher incidence of kidney dysfunction (93.8% vs. 77.3%, P = 0.048). The severity of stroke symptoms correlated with declining eGFR, both at presentation and after seven days. Reduced eGFR was identified as an independent predictor of poor stroke outcomes, with an odds ratio of 0.955 (95% CI: 0.924 - 0.986, P = 0.005) [24].

Ischemic stroke patients in our study had a 17% lower likelihood of death compared to hemorrhagic stroke patients (OR = 0.17; 95% CI: 0.06 - 0.50). Each additional year of age increased stroke-related mortality risk by 6% (OR = 1.06; 95% CI). Hemorrhagic stroke was linked to significantly higher overall mortality compared to ischemic stroke, with a hazard ratio of 1.564 (95% CI: 1.441-1.696) [25]. Bhattacharjee K et al. also reported higher mortality in hemorrhagic stroke patients (42.31%) than in ischemic stroke patients (13.51%) [26]. While hemorrhagic stroke patients had longer hospital stays, this difference was not statistically significant (P = 0.148). A similar study reported an average hospital stay of 9.24 ± 1.14 days for ischemic stroke patients and 11.21 ± 1.65 days for hemorrhagic stroke patients, with no statistically significant difference [27].

The modified Rankin Scale (mRS) was used to assess functional status at admission, on day 7, and at discharge. Hemorrhagic stroke patients had higher mortality, whereas ischemic stroke patients exhibited more severe initial symptoms, indicated by higher mRS scores at admission. Despite this, ischemic stroke patients showed faster recovery and better functional outcomes by discharge. Mortality rates increased with age and were higher for hemorrhagic stroke compared to ischemic stroke [28].

Elevated serum creatinine and low eGFR in our study were strongly associated with mortality, with a P-value of 0.0001. In a study by Jiang F et al., which included 381 stroke patients, the risk of acute kidney injury (AKI) was heightened by higher NIHSS (National Institutes of Health Stroke Scale) scores (OR 1.136, 95% CI: 1.074-1.202, P < 0.001), higher APACHE II scores (OR 1.107, 95% CI: 1.004-1.22, P = 0.042), and hypertension (OR 2.346, 95% CI: 1.244-4.426, P = 0.008) [29]. AKI patients had a significantly higher 28-day mortality rate compared to non-AKI patients (53.9% vs. 19.2%, P < 0.001) [29].

In our study, diagnostic performance testing revealed that a creatinine threshold of 1.495 mg/dL was optimal for predicting mortality risk in stroke patients, with a sensitivity of 69.6%, specificity of 81.8%, positive predictive value of 93.4%, and negative predictive value of 41.9%. For eGFR, the threshold was 48.5 mL/min, with a sensitivity of 86.4%, specificity of 69.5%, positive predictive value of 43.2%, and negative predictive value of 95.0%. Serum creatinine and eGFR can thus serve as useful biomarkers for assessing mortality risk in stroke patients. A study of 672 patients with non-traumatic subarachnoid hemorrhage found that serum creatinine was an independent prognostic indicator for all-cause mortality within 30 days of admission (OR 2, 95% CI: 1.18-3.41, P = 0.01) [30].

A meta-analysis of 168 studies, which included 5,611,939 participants and 115,770 stroke outcomes, found that an eGFR below 60 mL/min per 1.73 m² was associated with an increased risk of stroke (RR = 1.73; 95% CI: 1.57-1.90, P < 0.001) across 85 studies (3,417,098 participants and 72,996 strokes). However, there was substantial heterogeneity among the studies (P < 0.0001; I² = 78.5%) [31].

Strengths and limitations

Our study had several limitations. First, we cannot completely rule out the possibility that some patients had unknown or unmeasured confounding factors that predisposed them to death. However, given the number of variables we considered and the multiple logistic regression analysis we performed to identify factors independently associated with mortality, the impact of such confounders is likely to be small. Additionally, the study was conducted in a single hospital, which may limit the generalizability of the results to all stroke patients, as standards of care vary between facilities. Moreover, the short follow-up period, which only tracked stroke outcomes from admission through discharge, limits the ability to assess long-term recovery or survival.

Despite these limitations, the prospective design allowed for real-time data collection, reducing the likelihood of recall bias and enhancing the reliability of our findings. Furthermore, the study had several strengths. Comprehensive data collection, including clinical, demographic, and laboratory data such as serum creatinine and eGFR, provided robust insights into the relationship between renal function and stroke outcomes. By focusing specifically on renal function, we were able to highlight the significance of serum creatinine and eGFR as potential predictors of mortality in stroke patients. The use of multivariable logistic regression with backward stepwise selection further strengthened the study by minimizing confounding factors and identifying independent predictors of mortality.

## Conclusions

In conclusion, our study underscores the significant impact of renal function on stroke outcomes, highlighting both serum creatinine levels and estimated glomerular filtration rate (eGFR) as critical predictors of mortality among stroke patients. The higher mortality risk associated with hemorrhagic stroke compared to ischemic stroke, alongside the observed correlation between impaired renal function and adverse outcomes, emphasizes the need for integrating renal assessment into stroke management protocols. Our findings align with existing literature suggesting that renal dysfunction is a key factor influencing stroke prognosis but also point to the necessity of further research with larger sample sizes to confirm and refine these associations. Effective monitoring of renal markers could enhance the early identification of at-risk patients and potentially improve therapeutic strategies, ultimately contributing to better patient outcomes in stroke care.
